# Can an evidence-based mental health intervention be implemented into preexisting home visiting programs using implementation facilitation? Study protocol for a three variable implementation effectiveness context hybrid trial

**DOI:** 10.1186/s13012-024-01402-7

**Published:** 2024-11-11

**Authors:** Elissa Z. Faro, DeShauna Jones, Morolake Adeagbo, Hyunkeun Cho, Grace Swartzendruber, Karen M. Tabb, S. Darius Tandon, Kelli Ryckman

**Affiliations:** 1 Department of Internal Medicine, University of Iowa Carver College of Medicine, 200 Hawkins Dr, Iowa City, IA 52242 USA; 2 Institute for Clinical and Translational Science, University of Iowa, Iowa City, IA USA; 3 Department of Biostatistics, University of Iowa College of Public Health, Iowa CIty, USA; 4 School of Social Work, University of Illinois at Urbana-Champaign, Urbana, IL USA; 5 Department of Medical Social Sciences, Northwestern University Feinberg School of Medicine, Chicago, IL USA; 6 Indiana University School of Public Health, Bloomington, IN USA

**Keywords:** Perinatal mental health, Context, Ethnography, Home visiting, Trial, Facilitation

## Abstract

**Background:**

Perinatal mental health conditions are the most common complication of pregnancy and childbirth (1 in 8 women). When left untreated, perinatal depression and anxiety adversely affects the entire family with pregnancy complications and negative outcomes including preterm birth, impaired mother-infant bonding, impaired lactation, substance abuse, divorce, suicide, and infanticide. Significant disparities persist in the diagnosis and treatment of perinatal depression and anxiety and these inequities are often intersectional. Preliminary research with stakeholders including community advisory boards, underrepresented and minority birthing people, and state departments of health, demonstrates the importance of social support as a mechanism for reducing disparities in perinatal depression, particularly in rural geographies. Home visiting programs (HVPs) can provide the social support needed to improve mental health outcomes in pregnant and postpartum women. Our project aims to explore the impact of context on the implementation of a mental health intervention, focusing on the lived experiences of diverse populations served by HVPs to reduce disparities in adverse maternal outcomes.

**Methods:**

Using implementation facilitation, our study will engage multilevel stakeholders (e.g., policymakers, front-line implementers, and intervention recipients) to adapt facilitation to integrate a maternal mental health intervention (i.e., Mothers and Babies) across two midwestern, rural states (Iowa and Indiana) with multiple HVP models. Given the complexity and heterogeneity of the contexts in which Mothers and Babies will be integrated, a three variable hybrid implementation-effectiveness-context trial will test the adapted facilitation strategy compared with implementation as usual (i.e., standard education) and will assess contextual factors related to the outcomes. Using an evidence-based implementation strategy that tailors implementation delivery to the needs of the specific populations and context may improve fidelity and adoption, particularly in rural states where residents have limited access to care.

**Discussion:**

The immediate impact of this research will be to show whether adapted facilitation can improve the uptake and fidelity of Mothers and Babies across multiple HVP models and thus positively affect depressive symptoms and perceived stress of recipients. Our implementation protocol may be used by researchers, practitioners, and policy makers to better integrate evidence-based interventions into diverse contexts, leading to more equitable implementation and improved health outcomes.

**Trial registration:**

ClinicalTrials.gov Identifier: NCT06575894, registered on August 29, 2024 https://clinicaltrials.gov/study/NCT06575894?id=NCT06575894&rank=1.

Contributions to the literature

- There are few published protocols for a three variable implementation effectiveness context trial where context is the variable of interest.
- Our study will provide evidence for the impact of context on the implementation of a mental health intervention to provide action-based and impactful data that focuses on the lived experiences of the diverse populations served by home visiting programs in rural settings.
- Our implementation protocol may be used by researchers, practitioners, and policy makers to better integrate evidence-based interventions into diverse contexts, leading to more equitable implementation and improved health outcomes.
- Our theoretical and methodological approach to understanding context as the variable of interest in pragmatic implementation trials may be used broadly across international contexts, for example when adapting an intervention developed in one country to another.

## Background

Perinatal (during pregnancy and up to 1 year after birth) depression and anxiety are common medical complications of pregnancy contributing to a rising maternal mortality rate. When women experiencing anxiety and mild depression are included, the estimated prevalence of perinatal mental health conditions may be as high as 25% to 50% [1]. Moreover, the COVID-19 pandemic has exacerbated the mental health crisis across the globe, increasing the prevalence of anxiety and depression by 25% [2]. When left untreated, perinatal depression adversely affects the entire family with pregnancy complications and negative outcomes including preterm birth, impaired mother-infant bonding, impaired lactation, substance abuse, divorce, suicide and infanticide [1, 3–7]. Research suggests that only half of women with antenatal depression are identified, and only about 14% receive treatment [8]. This is particularly noticeable in data from the 2003–2007 National Violent Death Reporting System, where the maternal mortality rate from pregnancy-associated suicide was higher than reported mortality rates for hemorrhage/placenta previa or eclampsia and preeclampsia [9]. Therefore, addressing perinatal depression is of critical public health importance and could significantly lower maternal mortality rates due to pregnancy-associated suicide.

Significant racial/ethnic, rural/urban, and socioeconomic status (SES) disparities exist in the diagnosis and treatment of perinatal depression and anxiety. While some studies have found higher rates of perinatal depression and lower rates of treatment in Black, Hispanic, low-SES, and rural women, these inequities are often tied to one another [10, 11]. For example, previous research examined rural–urban differences in the risk of perinatal depression among 17,229 women who participated in the Pregnancy Risk Assessment Monitoring System survey [12]. We found that depression during pregnancy was 21% higher among rural compared with urban-residing women but that this risk is attenuated after adjusting for education, insurance coverage, and Women, Infants, and Children Program (WIC) participation, suggesting that socioeconomic factors in rural areas explain part of this disparity [12]. Intersectional identities face additional disparities; the Federal Office of Rural Health Policy (FORHP)-funded Rural Health Research Centers showed that rural counties with majority Black, Indigenous, and People of Color (BIPOC) populations have less access to local prenatal care and perinatal mental health services than do majority-White rural counties [13]. Although all rural counties studied had limited access to evidence-based family-centered models of care, this disparity was more pronounced for majority-BIPOC counties. Majority-BIPOC counties also had less access to doula care and postpartum peer support groups [13]. Largely rural states like Iowa and Indiana rank 23^rd^ and 42^nd^, respectively, on overall measures of mental illness prevalence and access to care [14]. This correlates with the maternal mortality rate, which is higher in Indiana (43.6 deaths per 100,000) than in Iowa (18.3 deaths per 100,000) [15].

Home visiting programs (HVPs) can provide the social support needed to improve mental health care outcomes for perinatal women. By enabling early detection and intervention, social support can mitigate the negative outcomes of perinatal depression [16]. A recent scoping review showed HVPs have been used worldwide to provide mental health screening, psychoeducation, case management, and social support through weekly or monthly home visits made by nurses, social workers, paraprofessionals, or trained volunteers [16]. Community-based HVPs are a practical and evidence-based intervention that can improve maternal and child health outcomes [17]. The same risk factors that qualify a woman for home visiting (e.g., low socioeconomic status, low social support, single or teen mother, inadequate prenatal care, low maternal education) are also risk factors for perinatal depression [16]. Moderate to severe levels of depressive symptoms are found in 45–50% of HVP recipients and home visitors report that clients with depression are more difficult to engage with during visits [18, 19]. Therefore, a need exists to specifically address perinatal mental health in community-based HVPs to ensure maximum benefit of home visiting services. The review by Tabb et al. identified 12 intervention studies conducted during the perinatal period and delivered by HVPs to improve postpartum depressive symptoms; these studies showed improvement in depressive symptoms from baseline to follow-up. Studies show that HVPs can be used to deliver mental health services without relying on the already-taxed mental health care system but produced mixed results due to research design factors and challenges with implementation [16, 20].

One such intervention that has proven to be particularly effective is Mothers and Babies (MB). MB is a well-supported, evidence-based cognitive behavioral intervention with demonstrated efficacy and effectiveness in reducing depressive symptoms and preventing perinatal depressive episodes among perinatal women [2, 11, 12]. Randomized controlled trials have shown that MB can prevent the onset of major depression, reduce depressive symptoms, and improve mood management in perinatal women. The most recent of these trials assessed the MB intervention one-on-one as delivered by trained lay health workers conducted in the setting of HVPs [18, 21–24]. In a type 2 hybrid implementation-effectiveness trial, even though less than 50% of eligible pregnant people received MB, participants who received at least one session of the MB intervention had better scores on the Beck Depression Inventory II (BDI) and the Perceived Stress Scale (PSS) than did control participants. While results showed the intervention was most effective when the participants received the full dose, only 34% of participants received MB without fidelity-inconsistent adaptations [25]. They concluded using evidence-based strategies that tailor implementation to the needs of specific populations and context could improve fidelity and adoption.

**Fig. 1:**
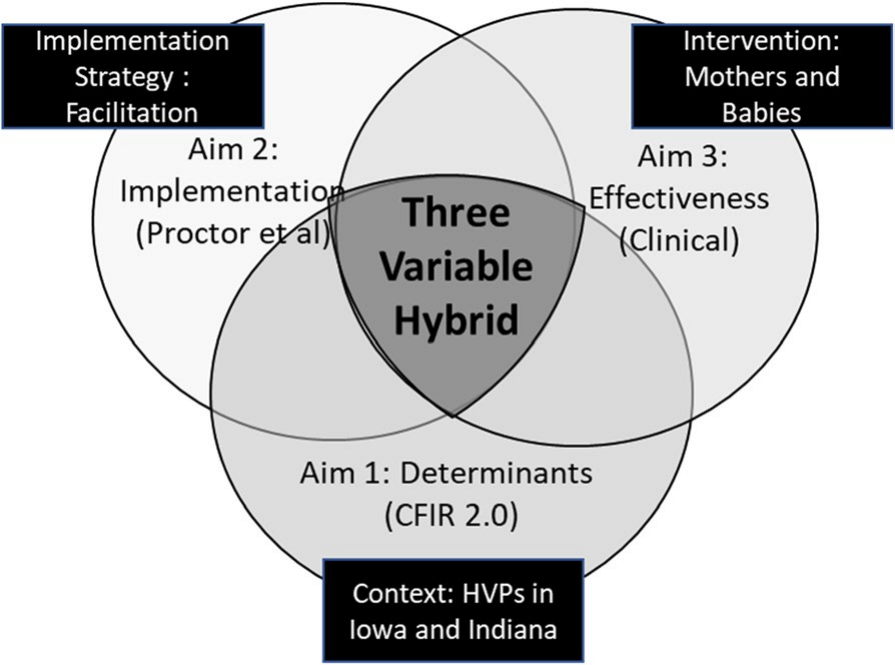
Conceptual model with i-PARIHS implementation framework

Anthropological ethnography and hybrid trial designs can provide the structure to ensure context-specific implementation. Anthropological ethnography can provide a deeper, richer, and more nuanced understanding of how minoritized populations experience embedded, structural, and systemic aspects of health and healthcare. Context is the explicit focus of ethnography and can ensure that our research not only includes previously underrepresented groups but prioritizes and amplifies their voices over those whom the system has historically privileged. MB is a maternal mental health intervention that has been proven efficacious, [21] and its implementation has been studied in a hybrid trial [25]. The results of these trials have explicitly concluded that better understanding of context, better engagement with stakeholders, and more thoughtful and granular analysis of differential delivery and uptake by diverse populations, is needed to effectively implement MB for all members of priority populations [25]. Three-variable hybrid designs expand the current hybrid implementation effectiveness designs by providing the structure to explicitly consider context as a third independent variable together with the intervention and implementation strategy [26]. The MB studies thus far demonstrate that understanding the effects of contextual determinants (e.g., funding, program, population, etc.) is the missing piece connecting previous implementation and effectiveness work on the intervention and implementation strategy to equitable implementation for all populations across variable contexts (Fig. 1) [26].

We will conduct a pragmatic cluster randomized trial to test the implementation effects of MB within HVPs on mental health outcomes (depressive symptoms and perceived stress). Given the complexity and heterogeneity of the contexts in which MB will be integrated, a hybrid implementation-effectiveness-context trial will test the adapted facilitation strategy compared with implementation as usual (i.e., standard education and ongoing technical assistance) and will assess contextual factors related to the outcomes. This study will address the following specific aims:

- Specific Aim 1: Adapt implementation facilitation to support uptake of MB using stakeholder input.
- Specific Aim 2: Analyze the effects of the adapted facilitation on implementation outcomes (i.e., adoption and fidelity) [26] using mixed rapid ethnographic methods (i.e., implementer and patient questionnaires, interviews, focus group discussions (FGD), observations, and geospatial data) [27, 28].
- Specific Aim 3: Determine the effect of adapted facilitation on the clinical outcomes: severity of depressive symptoms and perceived stress.

## Approach

Our multimethod data collection and analysis will be theory-driven by key concepts of the integrated Promoting Action on Research Implementation in Health Services (iPARIHS), [29] the updated Consolidated Framework for Intervention Research (CFIR 2.0), [30] and Proctor et al., [31] and more broadly by our overarching commitment to reproductive justice and birth equity [32, 33]. Previous research has established the evidence base for the intervention; [25, 34] the challenge is better implementation that fully engages stakeholders and uses a context-responsive strategy. We chose iPARIHS because context is a critical component and facilitation is its main strategy to respond to the specific context and setting of implementation [29, 35–37]. As a determinant framework, CFIR 2.0 will provide the structure for the stakeholder conversations in Aim 1; the updated framework centers recipients and reprioritizes relationships between multilevel stakeholders [30]. Additionally, we will use Proctor et al.’s implementation outcomes to frame our work in Aims 2 and 3 so we can disentangle core components of the intervention, how to adapt the implementation strategy, and how context may impact these differently [38]. The hybrid type 2 study of MB used RE-AIM, [25] but Proctor et al.’s more granular measures will clarify how certain contextual elements mediate or moderate implementation outcomes [26].

### Context: home visiting programs

Indiana and Iowa have HVPs funded by both the federal HRSA Maternal, Infant, and Early Childhood Home Visiting (MIECHV) program and the state. In Iowa, MIECHV supports four evidence-based HVPs: Healthy Families America (HFA), Early Head Start-Home-Based Option, Nurse Family Partnerships (NFP), and Parents as Teachers in 9 rural and 4 non-rural counties. State and MIECHV HVPs combined serve 92 of 95 counties in Iowa. In Indiana, MIECHV supports HFA and NFP in 6 non-rural counties. State and MEICHV HVPs combined serve all 92 counties in Indiana with 73% having more than one HVP available.

### Intervention: Mothers and Babies (MB)

MB is a well-supported, evidence-based cognitive behavioral intervention with demonstrated efficacy and effectiveness in reducing depressive symptoms and preventing perinatal depressive episodes among perinatal women [21, 25, 39]. MB is a 9-session intervention with each session designed to be delivered in 20–25 min. Program recipients are taught ways to increase thoughts and behaviors that lead to a positive mood state. The protocolized intervention has 3 modules that focus on (1) increasing enjoyable activities, (2) reframing harmful thoughts, and (3) increasing social support [39]. The MB training will present basic cognitive-behavioral theory, the MB 1-on-1 curriculum content, and the process for implementing the intervention in conjunction with delivering home visits (e.g., positioning of the MB session within the home visit agenda, alignment with the goals of the visit, modeling concepts for clients) [21, 25].

### Implementation strategy: facilitation

Implementation facilitation is a multifaceted, flexible, evidence-based implementation strategy; [40] that has been shown to improve implementation across interventions and contexts, including mental health [41, 42]. Facilitation is delivered as a bundled subset of a range of activities (a recent scoping review identified 32 discrete activities) that vary from goal-setting and problem-solving to training and marketing, depending on stakeholder needs [43]. This flexibility makes facilitation the most appropriate choice as the implementation strategy for our trial since it can be adapted to the patchwork of local contextual factors across the HVPs in Iowa and Indiana. Research team members will be trained and receive ongoing support as external facilitators through the Department of Veterans Affairs (VA) Quality Enhancement Research Initiative Training Hub’s Implementation Facilitation Training Program [35].

## Methods

### Formative evaluation

Our objective is to amplify the voices of pregnant/birthing people and to empower front-line implementers as active partners in our implementation research. We will conduct formative evaluation using participatory approaches to ensure engagement of stakeholders in the adaptation of the strategy as well as to improve adoption and fidelity. The context and evidence domains of iPARIHS in conjunction with specific determinant domains of CFIR 2.0 will provide the framework to better understand the contextual factors that impact implementation of MB and to account for different stakeholders’ perspectives as well as confirm the contextual variables to be measured in the trial. The context-specific adaption of implementation facilitation will be based on a shared understanding of the complex patchwork of HVPs in each state [44] and the current elements of psychosocial support. The research team will work with stakeholders to develop a shared understanding of the context of funding, policy, home visitors, training, recipients (as well as their families and communities), and the resources of existing HVPs in which MB will be implemented.

#### Key informant interviews

To gather context-specific information on the structural and social factors that may impact the participation of BIPOC birthing people in home visiting services, our research team will conduct brief key informant interviews with a purposefully sampled group of BIPOC birthing people. Interviews will focus on participants’ experiences, preferences, and barriers and opportunities to engage with HVPs with an interview guide informed by iPARIHS and CFIR 2.0.

#### Survey of HVPs

MIECHV services are tracked at the state level, but less is known about locally or state funded HVPs. We will conduct a brief survey of all HVPs within Iowa and Indiana through a REDCap survey asking each program to provide the numbers of 1) home visiting supervisors, 2) home visitors and 3) pregnant HVP recipients served each year. We will also assess which, if any, mental health services each program currently provides to determine initial interest our study.

### Stakeholder meetings

There will be two groups of HVP and maternal mental health stakeholders for each state who will meet within the same timeframe. To ensure all voices are heard equally and not affected by power dynamics, one group per state will consist of multilevel care delivery stakeholders and one group will consist of program recipient stakeholders. The care delivery group will include representatives from state departments of health (e.g., HVP Directors, home visiting epidemiologists, applied research coordinators, professional development coordinators), individual HVPs, and front-line implementers (e.g., home visitors, home visiting supervisors, social workers, embedded mental providers, doulas). The other group will consist of program recipient stakeholders (e.g., birthing people, families, and community members). All conversations will be audio recorded and research team members will take field notes.

We will use constructs of CFIR 2.0 organized by evidence, context, and facilitation (iPARIHS domains) that might influence differential uptake of MB, including local conditions and attitudes, assessing needs, setting goals, adaptations) [30, 36]. At the beginning of each meeting, the research team will briefly describe MB and the purpose of the meeting to participants. To identify the mechanisms of change responsive to specific contextual elements, we will present a review of annual MIECHV Program Reports, results of the HVP survey and key informant interviews, and relevant literature. The discussion will be framed by broad questions such as “What would it take to integrate MB into your current workflow?”; “What resources would you need to successfully facilitate implementation of MB?”; and “How can we best support you and your family during pregnancy?” to allow for breadth and depth of responses.

The transcripts of the discussions will inform the development of a context-adapted implementation facilitation through implementation mapping between the items and the standard elements of implementation facilitation [45]. For example, home visitors might identify a need for ongoing training in MB which would result in the facilitation adaptations to include additional education and capacity building sessions, while a program recipient might highlight stigma around mental health or need for participant education materials. These types of participant-identified issues will allow for adapted facilitation-trained home visiting supervisors to incorporate support and capacity building for the home visitors specifically to address those issues. We will reconvene the groups virtually to present the findings and propose the adapted facilitation for feedback and adjustment.

### Three variable implementation effectiveness context Cluster Randomized Control Trial (CRT)

**Fig. 2:**
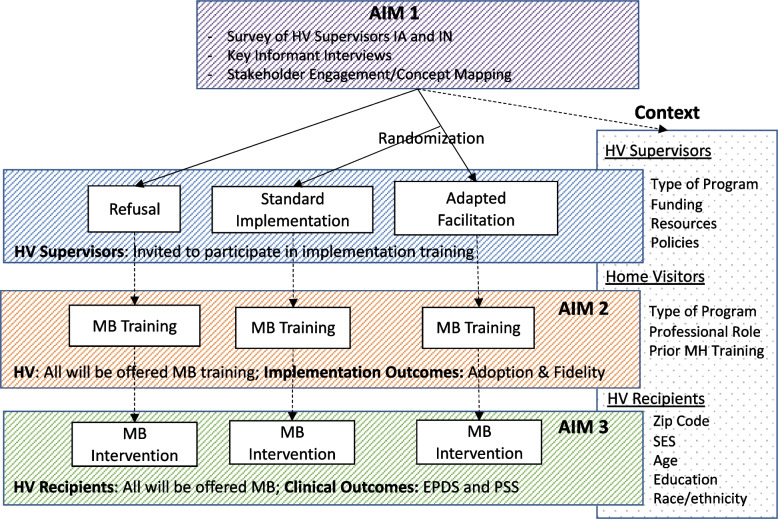
Study design

We propose a parallel two-arm CRT in Iowa and Indiana. Randomization will occur at the home visiting supervisor level (Fig. 2). We will randomly assign home visiting supervisors (Fig. 2) in a 1:1 plus adapted facilitation. Our 65% participation rate is based on a recent survey of home ratio to a control arm or an implementation strategy: 1) the *control arm* will receive standard MB training and implementation support and the 2) *implementation facilitation arm* will receive standard MB training visitor well-being in Iowa that had a response rate of 60%; we expect we will have a higher rate for our study as it is offering a training opportunity rather than completion of a survey. Outcome data to be used in Aims 2 and 3 will still be captured from home visitors and HVP recipients for supervisors who refuse to participate in the trial as MB training through administrative records. Therefore, adoption and fidelity outcomes will still be captured for home visitors and clinical outcomes on HVP recipients who are not part of the CRT.

### Three-variable hybrid design

Three-variable hybrid designs, while relatively new, seek to make explicit what many hybrid implementation effectiveness trials already do implicitly, which is take into account the role of contextual determinants in implementation [46–48]. Recent implementation research identifies the need to assess context explicitly as it impacts both implementation strategies and the intervention itself [49]. As the science moves toward more fully embedding equity into implementation work, it is critical that researchers incorporate context and stakeholder engagement more fully. Doing so will allow us to better measure and track the equitable delivery of evidence-based strategies and practices, in particular through a better understanding of structural factors (e.g., environmental exposures, systemic bias, etc.) [49–51]. The three-variable hybrid design is uniquely suited for the proposed implementation trial (expanding on a type 3) because 1) both the intervention and the implementation strategy are evidence-based; therefore, it is critical to test their relationship to context; and 2) the heterogeneity of contextual determinants (HVPs, funding, training, population, structural and environmental variables) requires explicit consideration.

#### Home visitors in control arm and outside the CRT

Home visitors in the control group and those not part of the CRT (i.e., declined participation) will receive standard MB training as developed and tested in RCT and hybrid type 2 trial, which involves 1.5-day group trainings. Following the training, control arm home visitors will receive standard support from their supervisors while those outside the trial will receive no support from their supervisors with respect to the MB intervention.

#### Adapted implementation facilitation arm

Home visitors in the adapted facilitation group will receive standard MB training plus adapted facilitation delivered by home visiting supervisors trained in adapted implementation facilitation. We will use facilitation adapted by the findings from Aim 1, and based on evidence that recognizes while dynamic and flexible, facilitation has core activities that have been identified as effective at different stages of implementation and for different types of implementers [52, 53]. The home visitor supervisors (internal facilitators) will receive a half-day training in adapted facilitation and ongoing self-driven and identified support from the external facilitators (i.e., research team members). In this pre- and early implementation, the external facilitators will employ core components of facilitation, including rapport and trust building, priority and goal setting, and clarifying roles and responsibilities, to support home visiting supervisors’ training as internal facilitators [54]. The training and ongoing support will be shaped by the findings from Aim 1 and may include issues such as: different needs and preferences of rural vs. urban HVP recipients; additional mental health resources for facilitators beyond MB-related issues; and if the HVP has existing mental health provision other issues the home visitors may face such as stigma, obstetric deserts, food insecurity, etc.

During implementation, the adapted facilitation-trained home visiting supervisors will draw upon core components of the strategy to support the home visitors in the front-line implementation of MB. These may include addressing resistance to change in specific home visiting contexts (e.g., rural) and supporting accountability by providing real-time feedback on implementation performance [54]. Additionally, internal facilitators will employ interactive problem-solving focused on supporting the home visitors to implement MB – working with pregnant people, challenges with resources, additional training needs, etc. – based on understanding the individual communities, contexts, and recipients’ and home visitors’ needs over the course of the project [35, 55, 56]. In the sustainment phase, the external and internal facilitators will continue to provide context-specific support as needed [54].

#### Randomization

Randomization will occur at the level of the home visiting supervisor. Supervisors will be randomized by study personnel who will not complete follow-up data collection. Using a sequence generated by study statistician, randomization will occur in permuted blocks of 4 and 6 and will be stratified by state. Allocation to intervention arm will remain concealed until inclusion and exclusion have been determined. This approach minimizes potential for experimenter and participant bias by protecting the randomization sequence and maintaining concealment of intervention allocation until the last moment. The home visiting supervisors will be aware of the trial and whether they are receiving training in adapted facilitation or in the control group. However, the home visitors and HVP recipients will not be aware there is an CRT in process so will not know to which treatment group they have been allocated or if they are not included in the cRCT.

### Trial data collection and analysis

The first objective is to analyze the uptake of MB by home visitors who receive adapted facilitation from their supervisors compared to those home visitors who receive standard support from their supervisors and those supervisors who refuse participation. This rapid ethnographic assessment (REA) will involve collecting qualitative and quantitative data (i.e., semi-structured interviews, observations, FGDs, questionnaires, and geospatial data) concurrently and iteratively and triangulating the results [27, 57, 58]. The questionnaires will allow us to measure implementation outcomes in real time and the qualitative data will provide a more nuanced understanding of why and how MB will be more readily adopted by certain home visitors, in certain HVP models, and whether delivery of the intervention varies for populations with different characteristics. As a secondary outcome, we will investigate which contextual factors identified in Aim 1 mediate or moderate the impact of adapted facilitation on implementation outcomes. The geospatial data will provide measurable indicators that will allow us to investigate the mediating or moderating effect of structural and systemic factors. We will also be able to examine factors that impact the choice of home visitor supervisors to participate in the CRT and how that impacts adoption and fidelity of MB among home visitors that are not part of the CRT.

### Qualitative component

#### Semi-structured interview and FGD guides

The research team will refine guides for the FGDs and interviews based on literature review, the iPARIHS and CFIR 2.0 frameworks, and any in vivo topics that may emerge from the Aim 1 formative evaluation. The guides will then be pilot tested with nonparticipants at partner organizations familiar with the topic and experiences of home visiting. This pilot testing will ensure that the questions are comprehendible and relevant to the Iowa and Indiana contexts. Research team members will conduct in-person or virtual interviews based on participant preferences and scheduling availability. The FGDs and interviews will be recorded, transcribed, and reviewed for accuracy. All transcripts will be analyzed using MAXQDA, secure qualitative data software [59].

#### Ethnographic observations

We will leverage existing meetings and site visits currently being conducted by IDHHS and IDOH with a combination of in-person and audio-recorded observations of: 1) weekly meetings between supervisors and visitors across all clusters in both arms to assess supervisor practice of adapted facilitation compared to standard implementation; 2) the existing biannual site visits conducted by the departments of health across a sample of models and staff, one of which is for monitoring, compliance, and needs assessment and the other for shadowing home visits, attending parent group meetings, etc. We will also attend or record monthly contractor calls, quarterly home visitor expert panel meetings, and quarterly participant advisory meetings.

##### Data analysis

The qualitative analysis team will conduct qualitative analyses using both deductive rapid and inductive in-depth methods. The analysis team will complete a *rapid ethnographic analysis* (e.g., template analysis, document review, etc.) throughout the data collection period to allow for real-time actionable adaptions to ongoing facilitation, and understand processes about MB and other main themes in a timely way [27, 57, 58]. Using a template developed with deductive, a priori iPARIHS and CFIR 2.0 domains particularly focused on inner setting variables including access to knowledge, culture, relational connections, compatibility, and structural characteristics, the analysis will also capture inductive, emergent themes. The analysis team will read a subset of transcripts to pilot the template and establish agreement and consistency among team members. Each transcript will be summarized independently by two members and then summaries will be reconciled until consensus is reached. The summaries will be entered into a matrix organized by the same domains and each domain will be analyzed vertically through analytic memos shared and discussed with the team. Ongoing feedback will be shared through external facilitation with HV supervisors (i.e., internal facilitators) to inform real-time adaptions to facilitation. In addition, the research team will conduct a *thematic analysis* that will allow for greater depth of analysis that can inform our understanding of the implementation outcomes by incorporating questions about differences in delivery and health outcomes for different populations in different contexts. The research team will read a subset of transcripts to generate a preliminary codebook, which the team will use to code a subset of three interviews independently and compare coding and examine agreement. We will conduct thematic analysis of each transcript independently to identify theoretical domains and constructs and to identify patterns. Once the threshold is reached, all subsequent coding will be performed by two coders.

### Quantitative component

#### Education and implementation questionnaires

We will use the validated baseline and follow-up education and retention questionnaire developed by the MB trainers [25] to assess knowledge and attitudes about MB and perceived self-efficacy among all the home visitors. We will also use a 5-item REDCap questionnaire for the home visiting supervisors (participating in the CRT) that will include closed questions about number of home visitors supervised, hours spent on supervision/facilitation, and open-ended questions about challenges and opportunities around supervision and additional support and resources needed. Fidelity will be assessed using the MB Home Visitor Fidelity Rating Form, which will be completed by the home visitors after each session.

#### Home visit documentation

We will leverage the DAISEY data system currently used by IDHHS which contains a home visit record form, completed within 48 h of a home visit [60]. We will develop a similar system to collect the same data from the Indiana HVPs.

##### Geospatial data

We will use the publicly available environmental justice screening and mapping tool (EJSCREEN), developed, and maintained by the Environmental Protection Agency (EPA). This nationally consistent dataset and methodology will allow us to calculate "EJ indexes" which assess the cumulative impact of environmental exposures (i.e., pollution, land use, green space, etc.) together with health and social vulnerabilities based on participant zip codes [61, 62]. EJ indexes will be our proxy variable for structural bias, which encompasses the complex interplay of systems that reinforce and perpetuate discrimination including housing, health care access, environmental exposures, etc. [63]. These geospatial indicators will provide quantifiable, intersectional contextual data as the critical and inextricable third variable in our model to help us understand how environmental and structural factors mediate or moderate both our implementation but also our clinical health outcomes [64]. These variables align with contextual variables with the CFIR outer setting domain, including financing, systemic conditions, policies and laws, and partnerships and connections [30].

In the analysis of cluster randomized trials, the generalize linear mixed model with the logit link function will be used to assess the effect of the adapted facilitation on implementation outcomes adoption and fidelity. We will include context specific variables (Table 1) in the model including type of HVP model, funding mechanism, EJ indices, and patient characteristics (race, age, geographic location) to identify the mediating or moderating effects. To account for the intracluster correlations, we include random effects for the level of home visitor and recipient in the model. Intent-to-treat analyses will be conducted on all those in the CRT. We will also examine implementation outcomes among home visitors that are not part of the CRT because their supervisor declined to participate and determine which contextual factors contribute to 1) participation in the trial; and 2) adoption and fidelity outcomes compared to the control and intervention arms of the CRT.

**Table 1:**
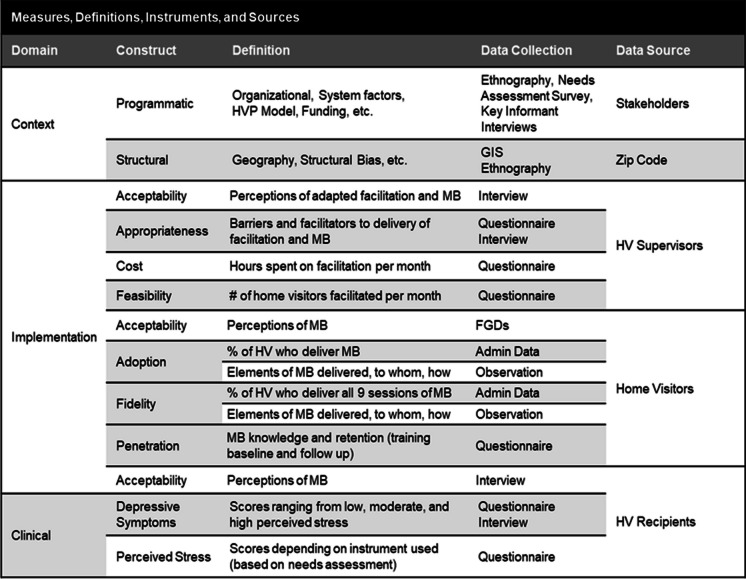
Measures, definitions, instruments, and sources

##### Power analysis

We defined cluster size (K) as the total number of HV recipients across home visitors within each supervisor. Given the K = 25, we calculated power based on different intracluster correlations (IC). We provided the total number of home visitor supervisors (N) required to achieve at least 90% power at a 5% Type I error rate (Table 2). We are well powered with the current number of home visitor supervisors estimated in each state to have ~ 100 per arm. Based on the work of Tandon et al. where the fidelity was ~ 35% we estimated the power needed to increase fidelity to 50%. The numbers were not drastically affected when changing K to 15 or 20.

**Table 2:** Power analysis for aim 2

IC	50% vs 35%
0.1	64
0.2	106
0.3	150
0.4	190
0.5	232

#### Clinical outcomes

We will test the hypothesis that recipients in the adapted facilitation arm have better clinical outcomes (e.g., lower depressive symptoms and perceived stress) compared to recipients receiving standard implementation of MB.

##### Data collection

Depression screening and perceived stress questionnaires. We will use similar baseline and follow-up surveys used in the effectiveness trials conducted by Tandon et al. We will harmonize with current practices being utilized among home visiting programs for depression and stress screening. We will ask that home visitors deliver both the EPDS and the Perceived Stress Scale (PSS), which is a 4-item questionnaire to their home visiting recipients as part of standard practice.

##### Statistical analysis

In the analysis of cluster randomized trials, the linear mixed model will be used to assess the effect of the adapted facilitation on each of two outcomes depressive symptoms and PSS at month 6 controlling for the baseline outcome. We will include the context specific variables mentioned in Aim 2, including the EJ indexes, and the random effects for the level of home visitor and recipient in the model. Intent-to-treat analyses will be conducted on all those in the CRT. We will also compare EPDS and PSS outcomes of those not in the CRT to both the control and intervention arms and identify the contextual factors that affect these outcomes.

##### Power analysis

We provided the total number of home visiting supervisors (N) required to achieve at least 90% power at a 5% Type I error rate (Table 3). We based our power calculations on the Tandon et al. study which found those receiving MB had Beck Depression Inventory II (BDI) and PSS scores that were 1.014 and 0.639 lower than the control group that did not receive the intervention. Because in this study all groups are receiving the intervention, we hypothesize that the adapted facilitation group will have depressive symptom scores and PSS scores that are lower (by approximately half) to the original study. We are well powered at all IC’s to detect the difference in depressive scores and at minimal IC’s for PSS.

**Table 3:** Power analysis for aim 3

	Effect Size	
IC	EPDS (0.5)	PSS (0.3)
0.1	48	128
0.2	80	218
0.3	112	308
0.4	144	398
0.5	176	488

##### Data triangulation

We will interrogate relationships between administrative, qualitative, quantitative, and geospatial data with a focus on understanding local perspectives on the variables to inform our analysis. We will develop preliminary matrices in our initial review of the data and will conduct pattern analysis to capture convergent, latent themes across all data sources. During this phase of analysis, our full research team will meet frequently to integrate qualitative and quantitative data using side-by-side comparisons of the qualitative data and joint displays, which include qualitative themes and selected dimensions from the quantitative data. The resulting triangulation will identify the effects of structural and programmatic determinants on both the fidelity and adoption of the intervention and then more clearly disentangle how contextual determinants impact clinical outcomes for program recipients. We will use participatory interpretation with our stakeholders by explaining our methods, presenting them with the results*,* and applying meaning together [65, 66]. For example, we will show relationships between lack of resources and fidelity and ask, ‘This is happening in your program/community, why do you think that is?’. By interpreting the results together, we will be able to disseminate findings quickly and effectively to relevant stakeholder groups in the forms of public presentations, state reports, white papers, conference papers and manuscripts in peer-reviewed journals.

## Discussion

Prioritizing user preferences to drive implementation is essential for the sustainability of mental health interventions that improve not only maternal well-being but that of the whole family. Resources available from patchwork funding sources are scarce given the multitude of other priority issues and unexpected pandemics; solutions that are responsive to local conditions are crucial. Implementation studies that are co-developed with stakeholders with the goal of sustainability can expedite the translation of research findings into practice. By understanding the role of specific contextual variables together with the effectiveness of the intervention and the best strategies for implementation, stakeholders can quickly scale up to new contexts. The next steps will be to provide the MB team with information about adapting to new contexts. Additionally, these study findings will inform the process for implementing future evidence-based practices into often heterogenous public health programs to improve health equitably for all populations. Finally, our theoretical and methodological approach to understanding context as the variable of interest in pragmatic implementation trials may be used broadly by researchers, implementers, and policy makers across international contexts, for example when adapting an intervention developed in a high-income country to low- or middle-income countries.

### Limitations

It is possible that we may not enroll enough home visitors or supervisors to obtain maximum power. The original effectiveness trial trained 473 home visitors with 51% delivering at least one MB session and completing the follow-up survey. Although the CRT examines home visiting supervisors delivering adapted facilitation, we estimate collecting administrative outcome data on all home visitors and HVP recipients, regardless of supervisor participation in the trial. We can alternatively randomize directly at the home visitor level, which would substantially increase our power to detect the effects. The external facilitators would then work with groups of home visitors delivering the adapted facilitation and home visitors, not the supervisors, would be randomized to the adapted facilitation intervention.

## Conclusion

The overall goal of this study is to engage with community members to improve the equitable delivery and uptake of a maternal mental health intervention, MB, into preexisting HVPs in Iowa and Indiana, which is essential to reduce unacceptable disparities in maternal morbidity and mortality. We will accomplish this overall goal through 1) needs assessment of contextual factors affecting facilitation as an implementation strategy and the integration of MB into current programs and models; 2) analyzing the effects of context and adapted facilitation on implementation outcomes (primarily adoption and fidelity), and 3) exploring our hypothesis that recipients in the adapted facilitation arm of the trial will have better clinical outcomes.

## Data Availability

The datasets used and/or analyzed in the current study will be available on the NIMH Data Archive.

## Abbreviations

BIPOC: Black, Indigenous, People of Color
SES: Socioeconomic status
HVP: Home visiting programs
MB: Mothers and Babies
IDHHS: Iowa Department of Health and Human Services
IDOH: Indiana Department of Health
CFIR 2.0: Consolidated Framework for Implementation Research
i-PARIHS: Integrated Promoting Action on Research Implementation in Health Services
RE-AIM: Reach, Effectiveness, Adoption, Implementation, and Maintenance
HRSA: Health Resources and Services Administration
MIECHV: Maternal, Infant, and Early Childhood Home Visiting
NFP: Nurse Family Partnership
CRT: Cluster Randomized Control Trial
FGD: Focus group discussions
EJScreen: Environmental justice screening and mapping tool
EPA: Environmental Protection Agency
EPDS: Edinburgh Perceived Depression Scale
PSS: Perceived Stress Scale
